# Cell-free hemoglobin triggers macrophage cytokine production via TLR4 and MyD88

**DOI:** 10.1152/ajplung.00123.2023

**Published:** 2023-11-22

**Authors:** Kaitlyn R. Schaaf, Stuart R. Landstreet, Sangami Pugazenthi, Emily Y. Qian, Nathan D. Putz, Tatiana Siderova, Allison M. Owen, Julia K. Bohannon, Lorraine B. Ware, Julie A. Bastarache, Ciara M. Shaver

**Affiliations:** ^1^Division of Allergy, Pulmonary, and Critical Care Medicine, Department of Medicine, Vanderbilt University Medical Center, Nashville, Tennessee, United States; ^2^Department of Pathology, Microbiology, and Immunology, Vanderbilt University Medical Center, Nashville, Tennessee, United States; ^3^Department of Anesthesiology, Vanderbilt University Medical Center, Nashville, Tennessee, United States; ^4^Department of Cell and Molecular Biology, Vanderbilt University, Nashville, Tennessee, United States

**Keywords:** acute lung injury, cell-free hemoglobin, inflammation, macrophages, TLR4

## Abstract

Cell-free hemoglobin (CFH) is elevated in the airspace of patients with acute respiratory distress syndrome (ARDS) and is sufficient to cause acute lung injury in a murine model. However, the pathways through which CFH causes lung injury are not well understood. Toll-like receptor 4 (TLR4) is a mediator of inflammation after detection of damage- and pathogen-associated molecular patterns. We hypothesized that TLR4 signaling mediates the proinflammatory effects of CFH in the airspace. After intratracheal CFH, BALBc mice deficient in TLR4 had reduced inflammatory cell influx into the airspace [bronchoalveolar lavage (BAL) cell counts, median TLR4 knockout (KO): 0.8 × 10^4^/mL [IQR 0.4–1.2 × 10^4^/mL], wild-type (WT): 3.0 × 10^4^/mL [2.2–4.0 × 10^4^/mL], *P* < 0.001] and attenuated lung permeability (BAL protein, TLR4KO: 289 µg/mL [236–320], WT: 488 µg/mL [422–536], *P* < 0.001). These mice also had attenuated production of interleukin (IL)-1β, IL-6, and tumor necrosis factor (TNF)-α in the airspace. C57Bl/6 mice lacking TLR4 on myeloid cells only (LysM.Cre^+/−^TLR4^fl/fl^) had reduced cytokine production in the airspace after CFH, without attenuation of lung permeability. In vitro studies confirm that WT primary murine alveolar macrophages exposed to CFH (0.01–1 mg/mL) had dose-dependent increases in IL-6, IL-1 β, CXC motif chemokine ligand 1 (CXCL-1), TNF-α, and IL-10 (*P* < 0.001). Murine MH-S alveolar-like macrophages show TLR4-dependent expression of IL-1β, IL-6, and CXCL-1 in response to CFH. Primary alveolar macrophages from mice lacking TLR4 adaptor proteins myeloid differentiation primary response 88 (MyD88) or TIR-domain-containing adapter-inducing interferon-β (TRIF) revealed that MyD88KO macrophages had 71–96% reduction in CFH-dependent proinflammatory cytokine production (*P* < 0.001), whereas macrophages from TRIFKO mice had variable changes in cytokine responses. These data demonstrate that myeloid TLR4 signaling through MyD88 is a key regulator of airspace inflammation in response to CFH.

**NEW & NOTEWORTHY** Cell-free hemoglobin (CFH) is elevated in the airspace of most patients with acute respiratory distress syndrome and causes severe inflammation. Here, we identify that CFH contributes to macrophage-induced cytokine production via Toll-like receptor 4 (TLR4) and myeloid differentiation primary response 88 (MyD88) signaling. These data increase our knowledge of the mechanisms through which CFH contributes to lung injury and may inform development of targeted therapeutics to attenuate inflammation.

## Graphical abstract

**Figure:**
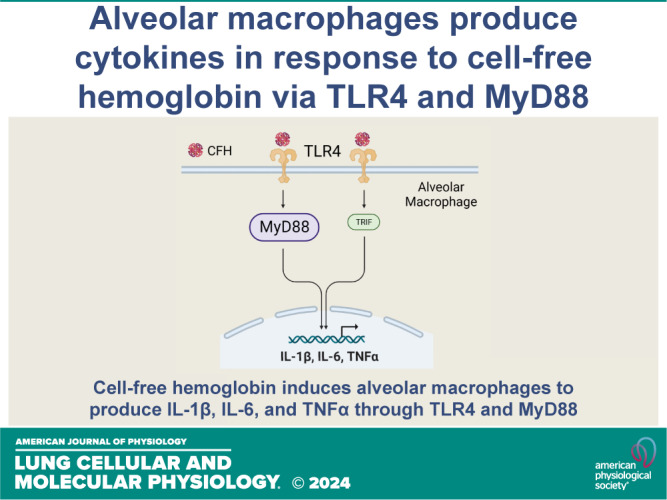

## INTRODUCTION

Acute respiratory distress syndrome (ARDS) is a form of severe lung injury characterized by intense lung inflammation, leading to disruption of the alveolar-capillary barrier, pulmonary edema, and respiratory failure (1). The pathophysiology of ARDS is complex (2) and an incomplete understanding of its pathogenesis has limited the development of targeted therapeutics. Previous studies from our group demonstrated that cell-free hemoglobin (CFH) is a key mediator of lung injury in ARDS. The majority of patients with ARDS have elevated CFH in the airspace (3) and intra-alveolar levels of CFH correlate with damage to the alveolar-capillary barrier in humans with ARDS and mice with experimental lung injury (4). Intratracheal CFH instillation in C57Bl/6 mice is sufficient to cause acute neutrophilic inflammation and disruption of the alveolar-capillary barrier in mice (4), establishing an effective animal model to study the mechanistic role of CFH in acute lung injury. Using this model, we previously showed that CFH can activate the NLR family pyrin domain containing 3 (NLRP3) inflammasome to contribute to airspace inflammation (5), but additional mediators of CFH-induced airspace inflammation are not well understood.

In this study, we aimed to delineate the role of Toll-like receptor 4 (TLR4) in CFH-induced lung inflammation. TLR4 is a major regulator of inflammation during acute lung injury and is critical for normal and pathogenic responses during both sterile and nonsterile lung injury. TLR4 recognizes pathogen-associated molecular patterns such as lipopolysaccharide (LPS) but can also respond to other damage-associated molecular patterns like oxidized lipoproteins (6, 7). After engaging its ligand, TLR4 recruits different adapter proteins, leading to the activation of either classical or alternative signaling pathways. The classical signaling pathway is activated at the plasma membrane when myeloid differentiation primary response 88 (MyD88) is recruited to TLR4, whereas the alternative pathway is activated when TIR-domain-containing adapter-inducing interferon-β (TRIF) is recruited by TLR4 on intracellular endosomes (6). Activation of these pathways culminates in the production of proinflammatory factors such as tumor necrosis factor (TNF)-α, interferon (IFN)-α, interleukin (IL)-1β, IL-6, and CXC motif chemokine ligand 1 (CXCL-1) (6, 8).

In the lung, TLR4 is most highly expressed on alveolar macrophages, but can also be induced on epithelial or endothelial cells during acute lung injury (9, 10). A critical role for TLR4 in acute lung injury has been shown in the models of pneumonia (11–14), hemorrhagic shock (15, 16), sepsis (17–19), bleomycin-induced pulmonary fibrosis (20), and hypoxia-induced acute lung injury (21). CFH has been shown to stimulate inflammatory responses in epithelial, endothelial, or immune cells in vitro. In addition to our work showing that CFH activation of the NLRP3 inflammasome in C57Bl/6 murine alveolar macrophages is partially dependent on TLR4 (5), we identified previously that CFH induces proinflammatory cytokine and chemokine production in cultured murine lung epithelial cells (4). Methemoglobin (the oxidized form of hemoglobin) induces proinflammatory cytokine production in human lung epithelial cells via NF-κB and MAPK (22), two signaling pathways that are downstream of TLR4 activation. Release of heme in a murine model of sickle cell disease induces TLR4-dependent inflammation in endothelial cells (23). Together, these studies indicate that CFH induces robust inflammation in the lung and suggest that TLR4 may be a candidate molecule that mediates these effects.

Thus, we hypothesized that TLR4 on alveolar macrophages mediates CFH-induced lung inflammation. To test this hypothesis, we used pharmacologic and genetic manipulations of TLR4 and its downstream signaling pathways to define the cell-specific and signaling pathway effects of TLR4 in vitro and in a murine model of CFH-induced acute lung injury.

## METHODS

### Mouse Strains

Male and female 8- to 16-wk-old mice deficient in TLR4 signaling (C.C3-TLR4^Lps-d^/J) (24) were bred in our animal facility and age-matched with the manufacturer-recommended control wild-type (WT) BALB/c control mice purchased from Jackson Laboratories (Bar Harbor, ME). Transgenic C57Bl/6 mice lacking TLR4 on myeloid cells were generated by crossing LysM.Cre^+/−^ mice (25) with TLR4^flox/flox^ mice on a C57Bl/6 background (26) (generously provided by Dr. Timothy Billiar). Loss of TLR4 was confirmed by genotyping, by RT-PCR for TLR4 in purified alveolar macrophages, and by immunohistochemistry (Supplemental Fig. S1). Cre^−/−^ TLR4^fl/fl^ C57Bl/6 littermates were used as controls. Male and female MyD88 knockout (MyD88KO, Stock 009088) (27) or TRIF knockout (TRIFKO, Stock 005037) (28) mice on the C57Bl/6 background were purchased from Jackson Laboratories along with WT C57Bl/6 controls. All mice were bred, housed, and treated according to approved Institutional Animal Care and Use Committee (IACUC) protocols.

### Animal Model of Cell-Free Hemoglobin-Induced Acute Lung Injury

Mice were anesthetized with isoflurane and 100 µg/mouse of cell-free hemoglobin (CFH; Cell Sciences, Canton, MA) dissolved in 100 µL of sterile phosphate-buffered saline (PBS) was instilled by direct intratracheal injection, as previously described (4). This dose of CFH was selected to mimic concentrations of CFH measured in the airspace of patients with ARDS (29). After 24 h, mice were euthanized. Bronchoalveolar lavage (BAL) was performed and lungs flash frozen and stored at −80°C until analysis. The concentration of inflammatory cells in BAL was manually enumerated and differentials determined after DiffQuick staining as previously described (4, 30, 31). BAL protein was measured by bicinchoninic acid (BCA) assay and BAL cytokines measured by multiplex electrochemiluminescent immunoassay [Meso Scale Discovery (MSD), Gaithersburg, MD] as described previously (5, 30, 31).

### Cell Purification and Culture Conditions

MH-S alveolar-like murine macrophages (initially isolated from BALB/c mice) were grown in RPMI-1640 supplemented with 10% fetal bovine serum, 1% penicillin/streptomycin, and 50 µM 2-mercaptoethanol according to ATCC guidelines. Primary alveolar macrophages were collected from naïve C57Bl/6 mice by three serial BALs with 900 µL of saline, adhered to tissue culture dishes for 1 h in culture media (RPMI with 10% FBS and 0.3 mg/mL l-glutamine) to enrich for macrophages, then aliquoted for experiments (5). Purity of macrophages was visually confirmed to be >95% for each experiment.

### In Vitro Exposure of Primary or Cultured Alveolar Macrophages to Cell-Free Hemoglobin

Cells were incubated with 0.1–1 mg/mL of CFH for 24 h, with this dose chosen to reflect clinically relevant levels of CFH in the airspace (29). MH-S cultured macrophages were incubated with human endotoxin-free CFH (Cell Sciences), whereas primary alveolar macrophages were incubated with CFH freshly purified from human blood in our laboratory (32); our laboratory switched from using commercially sourced CFH to isolation of CFH from normal human blood during the course of the studies reported in this manuscript. Both CFH preparations induced proinflammatory cytokines from macrophages to similar degrees (data not shown). After 6 or 24 h, conditioned media was collected, centrifuged at 400 *g* for 10 min to remove cell debris, and stored at −80°C until further analysis. Cytokine levels were measured in conditioned media by multiplex electrochemiluminescent immunoassay (Meso Scale Discovery).

### TLR4 Inhibition In Vitro

MH-S cells were exposed to 10 µg/mL of lipopolysaccharide (LPS; Sigma, St. Louis, MO) or 1 mg/mL of CFH (Cell Sciences) for 6 h in the presence or absence of TLR4 pathway inhibitor TAK-242 (3 µM, InvivoGen, San Diego, CA). DMSO was used for the vehicle control, with equivalent volume added to the media at the time of stimulation. Conditioned media was collected and cytokine expression measured as earlier. For cytokine mRNA expression, RNA was extracted from cell lysates using an RNeasy mini kit (Qiagen, Germantown, MD), cDNA was prepared using SuperScript VILO cDNA Synthesis kit (Thermo Fisher, Waltham, MA), then semiquantitative PCR was performed using primers from Thermo Fisher (5). Values were normalized to the mean expression in PBS-treated samples by ΔΔCt analysis (33) using the housekeeping gene GAPDH.

### Immunofluorescent Staining for TLR4

Five micrometers thick paraffin sections were cut, deparaffinized, rehydrated, and permeabilized with 0.25% Triton X-100 in PBS for 20 min at room temperature (RT). After washing with PBS and blocking with 5% BSA with 0.1% Triton X-100 for 1 h, tissues were incubated overnight with the primary antibody targeting TLR-4 (sc-30002, Santa Cruz) diluted 1:100 in PBS with 3% BSA and 0.1% Triton X-100, followed by incubation for 1 h at RT with donkey anti-rabbit Alexa Fluor 488-conjugated secondary antibody (A21206, Life Technology), diluted 1:500 in PBS with 1%BSA and 0.1% Triton X-100. All sections were counterstained by cover slipping the slides with ProLong Gold antifade reagent with DAPI. Negative control staining was also performed on separate sections with rabbit IgG substituted for primary antibody. Immunofluorescence images were obtained within 24 h using confocal microscopy (LSM880 Airy Scan, Zeiss). Images are shown in Supplemental Fig. S1.

### Statistical Analysis

Continuous variables between two groups were analyzed using Mann–Whitney *U* comparison for nonparametric data and Student’s *t* test for parametric data as appropriate and specified within figure legends. Comparisons of in vitro data across genotypes, treatments, and groups were performed by two-way ANOVA followed by Sidak’s multiple comparison. Analysis was performed using SPSS version 28 (IBM, Armonk, NY) and Prism version 9 (GraphPad Software, Boston, MA).

## RESULTS

### TLR4 Is a Key Regulator of Cell-Free Hemoglobin-Induced Inflammation and Permeability In Vivo

**Figure 1.**
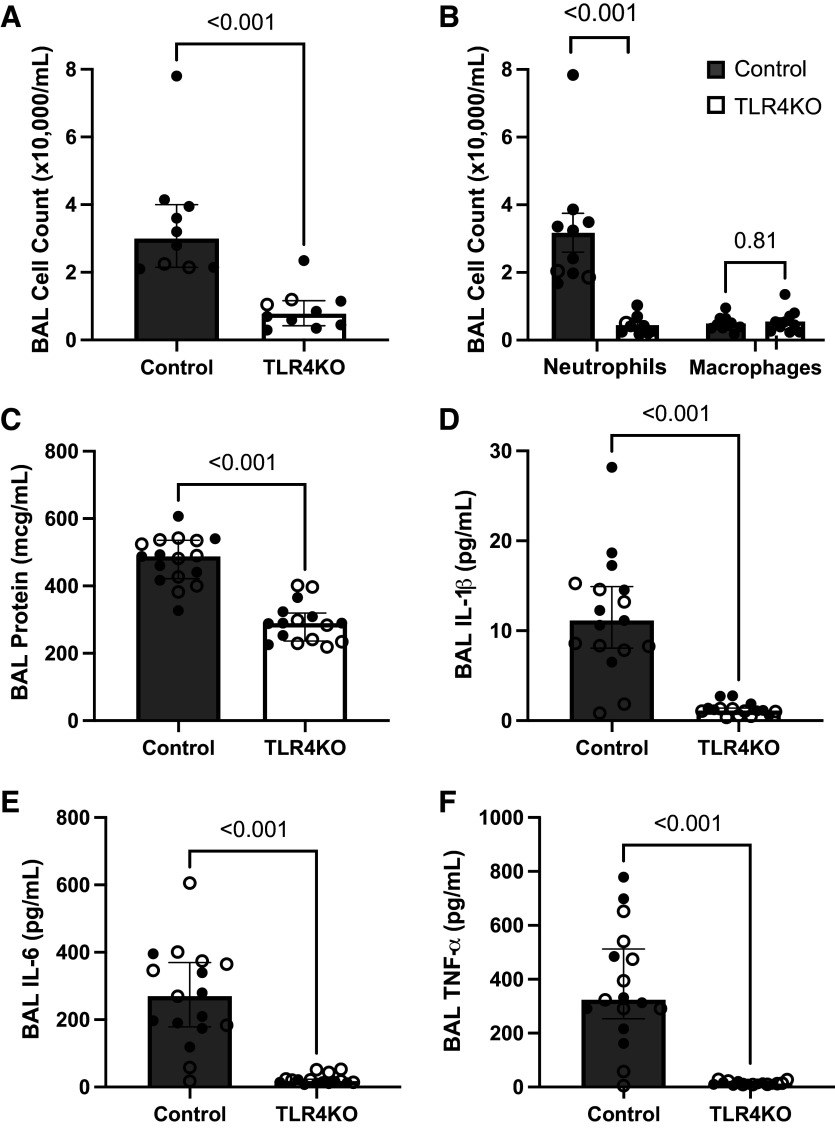
Toll-like receptor 4 (TLR4) is a key regulator of cell-free hemoglobin-induced acute lung injury. Mice lacking functional TLR4 knockout (TLR4KO) and wild-type BALB/c control mice were treated with intratracheal injection of cell-free hemoglobin (CFH, 100 µg, Cell Sciences) and samples collected after 24 h. Loss of TLR4 was associated with reduced inflammatory cell infiltration into the airspace (Control: *n* = 8 males, *n* = 2 females; TLR4KO: *n* = 8 males, *n* = 2 females) (*A*), reduced neutrophil influx (Control: *n* = 8 males, *n* = 2 females; TLR4KO: *n* = 8 males, *n* = 2 females) (*B*), and reduced disruption of the alveolar-capillary barrier as measured by bronchoalveolar lavage (BAL) total protein (Control: *n* = 8 males, *n* = 8 females; TLR4KO: *n* = 8 males, *n* = 8 females) (*C*). TLR4 was also required for upregulation of interleukin (IL)-1β (*D*), IL-6 (*E*), and tumor necrosis factor (TNF-α, *F*) in the airspace after exposure to cell-free hemoglobin as measured by ELISA (Control: *n* = 8 males, *n* = 9 females; TLR4KO: *n* = 8 males, *n* = 8 females). Graphs show individual data points with median depicted as a box and interquartile range as whiskers, closed circles are male animals, open circles are female animals, comparisons by Mann–Whitney *U* testing between genotypes.

We have previously shown that intratracheal administration of CFH to C57Bl/6 mice causes significant neutrophilic inflammation in the airspace and increases alveolar-capillary barrier permeability (4). To determine the role of TLR4 in CFH-mediated lung inflammation and barrier permeability, mice with a point mutation in TLR4 that abrogates TLR4-mediated signaling or WT BALB/c controls were exposed to intratracheal CFH for 24 h. In the absence of TLR4 signaling, influx of inflammatory cells into the airspace was impaired, with a 3.8-fold reduction in BAL total inflammatory cells (*P* < 0.001; Fig. 1*A*) due to a reduction in neutrophils (Fig. 1*B*). In addition, in the absence of functional TLR4, there was less disruption of the alveolar-capillary barrier as measured by BAL protein (*P* < 0.001; Fig. 1*C*). Mice lacking functional TLR4 also had less IL-1β, IL-6, and TNF-α in the airspace after CFH (*P* < 0.001 for each; Fig. 1, *D–F*), whereas production of CXCL-1, IL-10, and IL-12p70 were unchanged (data not shown). Together, these data show that TLR4 is a key mediator of CFH-induced lung inflammation and alveolar-capillary barrier permeability in vivo.

### TLR4 on Myeloid Cells Mediates Airspace Inflammatory Cytokines in Response to Cell-Free Hemoglobin

**Figure 2.**
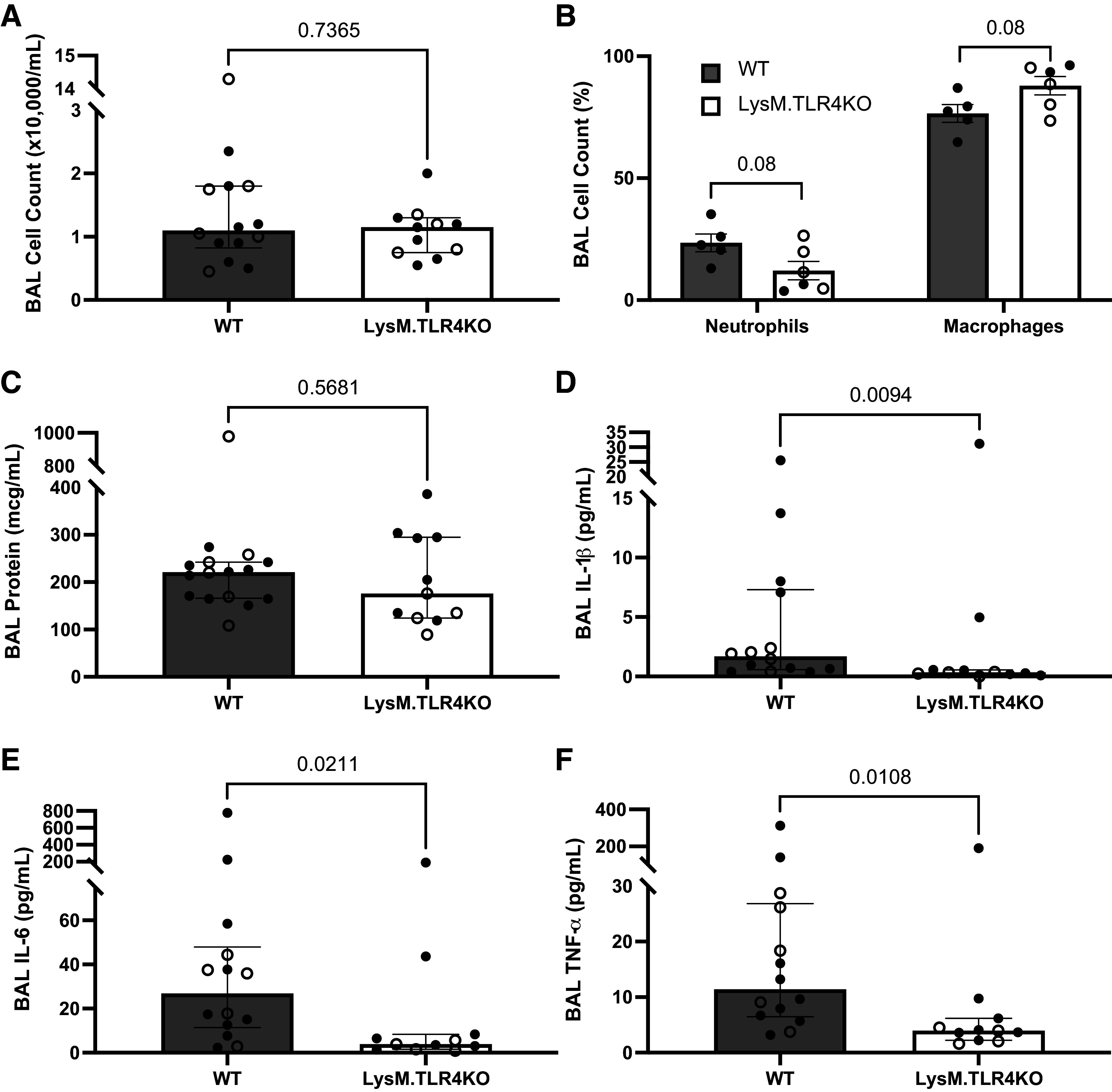
Toll-like receptor 4 (TLR4) on myeloid cells influences airspace inflammation in response to cell-free hemoglobin. LysM-TLR4 knockout (KO) mice or wild-type (WT) littermate controls on a C57Bl/6 background were treated with intratracheal cell-free hemoglobin (100 µg, Cell Sciences) and bronchoalveolar lavage (BAL) collected after 24 h. Deletion of TLR4 in myeloid cells was not associated with any significant change in total inflammatory cell infiltration into the airspace (WT: *n* = 8 males, *n* = 6 females; LysM.TLR4KO: *n* = 7 males, *n* = 4 females) (*A*), neutrophil influx (WT: *n* = 5 males; LysM.TLR4KO: *n* = 2 males, *n* = 4 females) (*B*), or disruption of the alveolar-capillary barrier as measured by BAL total protein (WT: *n* = 10 males, *n* = 6 females; LysM.TLR4KO: *n* = 7 males, *n* = 4 females) (*C*). Deletion of TLR4 was associated with reduced BAL levels of interleukin (IL)-1β (*D*), IL-6 (*E*), and tumor necrosis factor (TNF)-α (*F*) as measured by ELISA (WT: *n* = 9 males, *n* = 5 females; LysM.TLR4KO: *n* = 7 males, *n* = 4 females). Graphs show individual data points with median depicted as a box and interquartile range as whiskers, closed circles are male animals, open circles are female animals, comparisons by Mann–Whitney *U* testing between genotypes.

In the lung, TLR4 is predominantly expressed on myeloid cells: monocytes, macrophages, and neutrophils (9, 10). To test the specific contributions of TLR4 on myeloid cells to CFH-induced lung inflammation and permeability, we generated C57Bl/6 mice with targeted cell-specific deletion of TLR4 in the myeloid lineage (LysM-TLR4KO). LysM-TLR4KO mice as well as WT C57Bl/6 littermate controls were administered intratracheal CFH and samples were collected after 24 h. In this series of experiments, CFH-induced lung injury in WT mice was relatively modest and had a wide standard deviation. Myeloid cell TLR4 deletion had no significant impact on BAL inflammatory cell influx (Fig. 2, *A* and *B*), or BAL protein as a measure of alveolar-capillary barrier permeability (Fig. 2*C*). In contrast, myeloid cell TLR4 had modest effects on airspace production of IL-1β (*P* = 0.009), IL-6 (*P* = 0.021), and TNF-α (*P* = 0.011; Fig. 2, *D–F*), with lower levels of these inflammatory cytokines found in the absence of myeloid TLR4. These data show that myeloid TLR4 is necessary for the lung inflammatory cytokine response to CFH, but dispensable for CFH-induced inflammatory cell recruitment and injury to the alveolar-capillary barrier.

### Cell-Free Hemoglobin Induces Proinflammatory Cytokine Production in Primary Alveolar Macrophages

**Figure 3.**
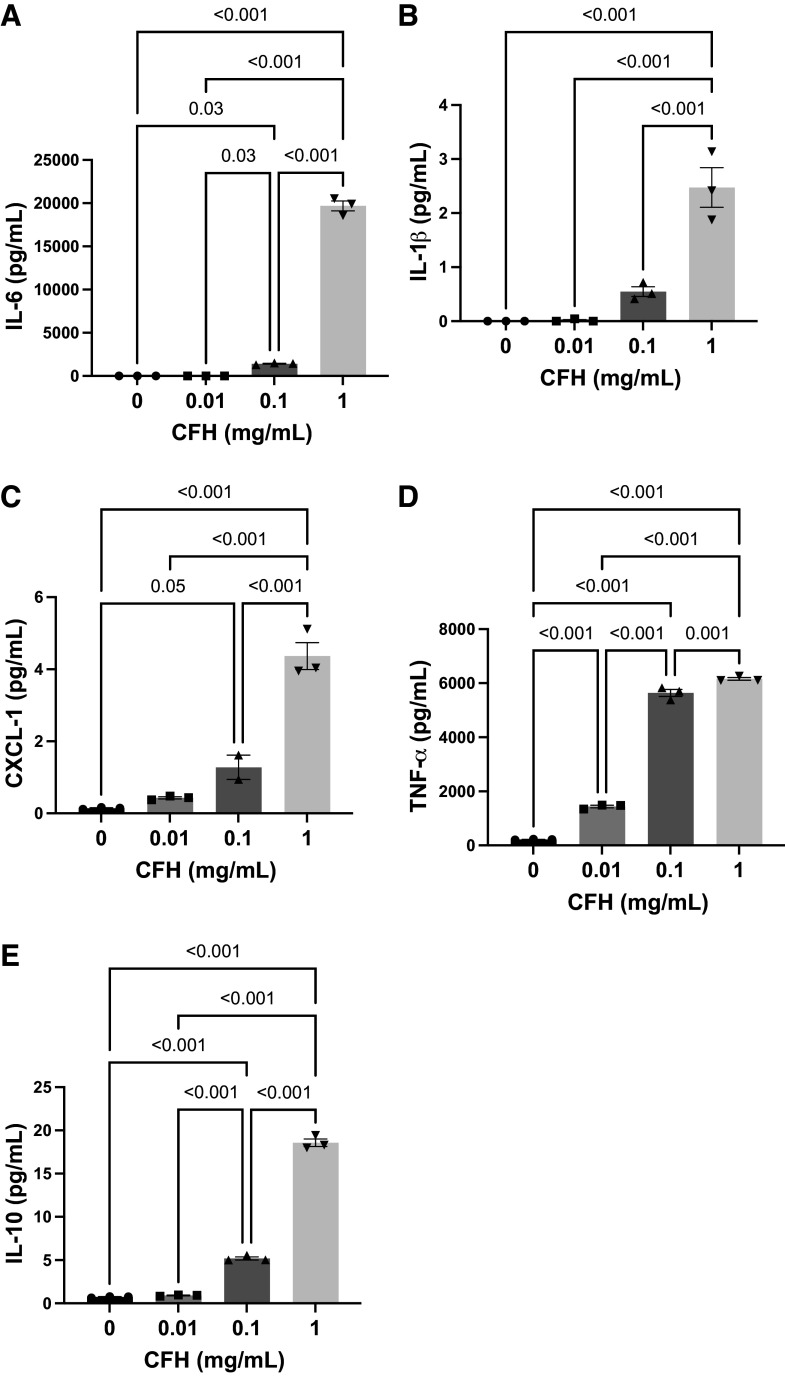
Cell-free hemoglobin (CFH) induces proinflammatory cytokine production in primary alveolar macrophages. Primary alveolar macrophages were purified from wild-type C57Bl/6 mice and exposed to CFH (0–1 mg/mL, freshly purified) for 24 h and cytokine expression in conditioned media assessed using multiplex electroluminescent immunoassays (Meso Scale Discovery). Production of interleukin (IL)-6 (*A*), IL-1β (*B*), CXC motif chemokine ligand 1 (CXCL-1, (*C*), tumor necrosis factor (TNF-α, *D*), and IL-10 (*E*) increased in a dose-responsive manner. *n* = 3 independent experiments with cells from male mice with multiple replicates each, graphs show individual data point for each day of experiment with mean depicted as a box and standard error of the mean as whiskers, comparisons using one-way ANOVA with post hoc Tukey testing for multiple comparisons.

We have previously demonstrated that CFH induces proinflammatory cytokine production in MLE-12 lung epithelial cells (4). To determine whether CFH causes similar proinflammatory responses in alveolar macrophages, freshly isolated primary alveolar macrophages from WT C57Bl/6 mice were exposed to increasing concentrations of CFH for 24 h and cytokine and chemokine production was measured in conditioned media. CFH increased the production of IL-6, IL-1β, CXCL-1, TNF-α, and IL-10 from macrophages in a dose-dependent manner (Fig. 3, *A–E*). These data show that macrophages generate proinflammatory cytokines in response to CFH.

### Proinflammatory Cytokine Production by Macrophages in Response to Cell-Free Hemoglobin Requires TLR4 Signaling

**Figure 4.**
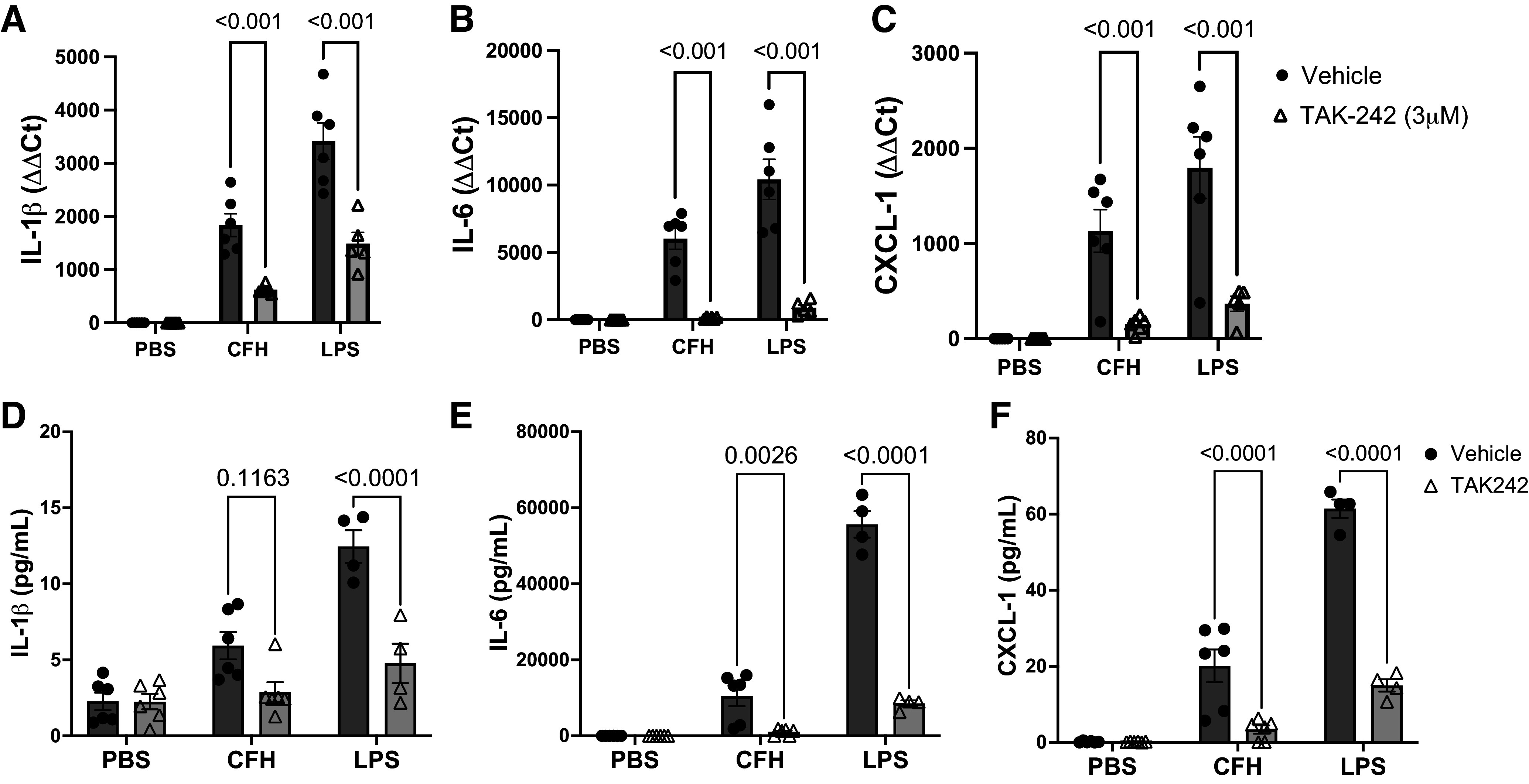
Cell-free hemoglobin (CFH) induces proinflammatory cytokine production in a TLR4-dependent manner. MH-S alveolar-like cultured macrophages were exposed to CFH (1 mg/mL, freshly purified) or lipopolysaccharide (LPS, 10 µg/mL) for 24 h in the presence or absence of the Toll-like receptor 4 (TLR4) signaling inhibitor TAK-242 (3 µM). Inhibition of TLR4 signaling prevented upregulation of interleukin (IL)-1β (*A* and *D*), IL-6 (*B* and *E*), and CXC motif chemokine ligand 1 (CXCL-1, *C* and *F*), as measured by RT-qPCR and ELISA, respectively. Inhibition of LPS-induced cytokine production is included as a positive control for TAK-242 inhibition. *n* = 6/group, graphs show individual data points with mean depicted as a box and standard error of the mean as whiskers, comparisons using two-way ANOVA with Sidak’s test for multiple comparisons.

Next, to determine whether pharmacologic inhibition of TLR4 in macrophages might also alter cytokines in response to CFH, we tested whether CFH induces inflammation in MH-S cells, an alveolar macrophage-like cell line. MH-S cells were exposed to CFH in the presence and absence of the TLR4 inhibitor, TAK-242. TAK-242 binds to the intracellular domain of TLR4, blocking the interaction of adaptor molecules with the receptor, thereby inhibiting signaling (34). Inhibition of TLR4 significantly impaired the expression of IL-1β, IL-6, and CXCL-1 after CFH exposure (Fig. 4, *A–C*). For IL-1β, TAK242 reduced induction by CFH by 66.2%, for IL-6 by 97.6%, and for CXCL-1 by 86.4% (*P* < 0.001 for each). We additionally assessed secreted protein levels of each cytokine (Fig. 4, *D–F*), confirming that pharmacologic inhibition of TLR4 markedly reduces cytokine production in alveolar macrophage-like cells in vitro. As a positive control for TAK-242 activity, we verified that TAK-242 inhibited LPS-induced inflammation. Together, these data show that TLR4 signaling is required for macrophage-mediated proinflammatory responses to CFH in vitro.

### Cell-Free Hemoglobin-Induced TLR4-Dependent Proinflammatory Cytokine Production Is Predominantly Mediated via MyD88 with Limited Contribution of TRIF

**Figure 5.**
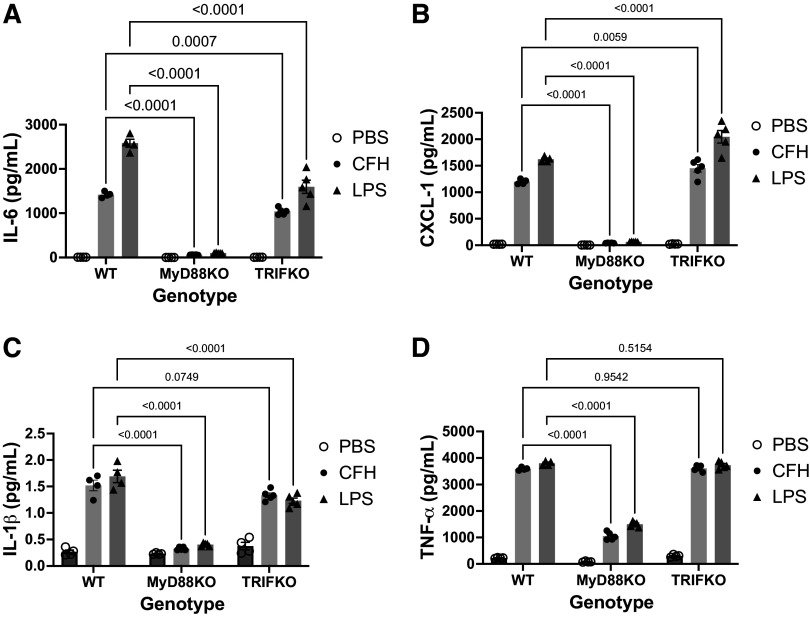
Cell-free hemoglobin (CFH) induced Toll-like receptor 4 (TLR4)-dependent proinflammatory cytokine production is regulated by both myeloid differentiation primary response 88 (MyD88) and TIR-domain-containing adapter-inducing interferon-β (TRIF). Primary alveolar macrophages isolated from wild-type (WT), MyD88 knockout (KO), or TRIFKO mice on the C57Bl/6 background were exposed to CFH (1 mg/mL, freshly purified) for 24 h and cytokine levels in conditioned media were measured by ELISA. Production of interleukin (IL)-6 (*A*), CXC motif chemokine ligand 1 (CXCL-1, *B*), IL-1β (*C*), and tumor necrosis factor (TNF)-α (*D*) was substantially reduced in the absence of MyD88, whereas only IL-6 was reduced in the absence of TRIF. In TRIFKO cells, CXCL-1 was increased and IL-1β and TNF-α were unchanged in response to CFH. *n* = 4 or 5 mice/genotype (all male due to availability from Jackson Laboratory), *n* = 4 or 5 replicates/treatment group, graphs show individual data points with mean depicted as a box and standard error of the mean as whiskers, comparisons by two-way ANOVA with Sidak’s test for multiple comparisons.

TLR4 has two primary signaling cascades mediated through the adapter proteins MyD88 and TRIF, which activate different transcription factors (6, 8). To determine which TLR4 signaling pathway drives the inflammatory response to CFH, primary alveolar macrophages from C57Bl/6 MyD88KO and TRIFKO mice were exposed to CFH ex vivo and cytokine production quantified. MyD88KO alveolar macrophages had markedly reduced expression and production of IL-6 (4% of WT levels, *P* < 0.001), CXCL-1 (4% of WT, *P* < 0.001), IL-1β (22% of WT, *P* < 0.001), and TNF-α (29% of WT, *P* < 0.001) (Fig. 5, *A–D*). TRIFKO alveolar macrophages had significantly reduced IL-6 (*P* < 0.001), but increased CXCL-1 (*P* = 0.006) and no change in IL-1β (*P* = 0.075) or TNF-α (*P* = 0.954). These data show that both the MyD88 and TRIF signaling pathways are involved in the cytokine response of alveolar macrophages to CFH, though MyD88 has a more pronounced effect.

## DISCUSSION

In this study, we aimed to determine the mechanistic role of TLR4 in airspace inflammation caused by CFH. Our results demonstrate that TLR4 is important for CFH-induced inflammatory cell influx to the lung, proinflammatory cytokine production in the airspace, and disruption of the alveolar-capillary barrier. Furthermore, we show that TLR4 on myeloid cells modestly reduces proinflammatory cytokine production in the airspace in response to CFH. Using pharmacologic approaches, we confirmed that TLR4-dependent signaling is required for CFH-induced cytokine production by cultured macrophages. In primary alveolar macrophages, we demonstrate that MyD88-dependent signaling is more dominant than TRIF-mediated signaling in response to CFH. These findings expand our knowledge of the mechanisms through which CFH contributes to airspace inflammation and identify the cell-specific signaling that is involved.

The key finding of the current study is that TLR4 affects airspace proinflammatory cytokine production in response to CFH. Previous in vitro studies showed that CFH induces proinflammatory cytokines via TLR4 in peritoneal macrophages (34), bone marrow-derived macrophages (35, 36), and microglia (37, 38). Heme, the iron-containing porphyrin moiety of CFH, also activated cytokines in cultured macrophages via TLR4 (39, 40). Our current study extends these in vitro data to show the cell-specificity of TLR4 modulation of CFH-associated cytokine production in the airspace. Despite myeloid TLR4 being important for maximal proinflammatory cytokine production in the airspace, we found that the absence of myeloid TLR4 did not reduce the overall influx of inflammatory cells into the injured airspace. This finding suggests that TLR4 on other cell types may be sufficient to promote neutrophil influx to the injured lung. Because our studies use mice and cells from both a C57Bl/6 background and a BALB/c background, our data support that CFH-induced inflammation depends on TLR4 in multiple murine genetic backgrounds. Our current findings also build on previous data and emphasize an important role for TLR4 in heme-mediated tissue injury. Heme is a component of CFH and previous studies have shown that TLR4 is important for the injurious effect of heme (41) in the models of hemolysis (42), intracerebral hemorrhage (43), sickle cell disease (23), and in a trauma/transfusion model of lung injury (41, 42). Because we have previously shown that hemin (the chloride salt of heme) was insufficient to cause airspace inflammation (4), we did not investigate heme in the current study. Overall, our data provide new information on the cell-specific role of TLR4 in the airspace in vivo in the injured lung.

Our findings indicate that MyD88 is the dominant signaling pathway for CFH-dependent cytokine production by C57Bl/6 primary alveolar macrophages ex vivo. This finding builds on previous studies of subarachnoid hemorrhage which show CFH activates MyD88-associated proteins IRAK1 and TRAF6 in microglial cells, increasing TAK1-dependent phosphorylation and leading to inflammatory neuronal cell death (37). The role of TRIF signaling in CFH-dependent cytokine production was more variable, affecting IL-6 and CXCL-1 but not IL-1β and TNF-α. It is possible that in the absence of TRIF, MyD88 is exclusively recruited to TLR4 and masks a potential contribution of TRIF to cytokine production. A greater role for MyD88 compared with TRIF in CFH-associated cytokine responses may suggest that TLR4 localization between the plasma membrane and intracellular membranes may be altered during CFH-induced injury, as MyD88 signaling occurs at the plasma membrane whereas TRIF signaling is associated with endosomal TLR4 (6). Understanding the signaling pathways that mediate CFH-TLR4-dependent cytokine production provides insight into how macrophages may be affecting airspace injury due to CFH and provides a foundation for future studies on macrophage-specific TLR4 signaling in vivo.

Deficient TLR4 signaling and genetic deletion of myeloid TLR4 had different effects on alveolar-capillary permeability in vivo. Mice with global absence of functional TLR4 on the BALB/c background had attenuated CFH-dependent lung permeability, whereas mice with myeloid deletion of TLR4 on a C57Bl/6 background had no change in CFH-dependent lung permeability. One explanation for this discrepancy could be that nonmyeloid TLR4 drives changes in alveolar-capillary barrier integrity. This explanation would be consistent with previous studies showing that TLR4 signaling is required for increased expression of leukocyte adhesion molecules in endothelial cells after exposure to CFH (44), for complement-associated damage of the endothelium during hemolysis (45), and for development of acute chest syndrome mediated by nonhematopoietic cells in a murine model of sickle cell disease (42). Previous research in intestinal TLR4 found a similar discrepancy between global and cell-specific TLR4 manipulation. In intestinal epithelial cells, LPS or alcohol activation of TLR4 via MyD88 disrupted tight junctions and increased intestinal barrier permeability (46, 47), whereas intestinal epithelial-specific knockout of TLR4 did not alter intestinal permeability in vivo (48, 49). Together with our data, this suggests that there may be redundant mechanisms among multiple cell types that alter the effects of TLR4 on barrier permeability. Another potential explanation for discrepant findings between genetic deletion and signaling-deficient TLR4 model could be that dysfunctional protein is still present on the cell surface in signaling-defective animals and could act as a decoy receptor. If this was true, CFH may bind a nonfunctional TLR4 and be sequestered away from other cells or signaling molecules. Finally, because our studies use mice on both a BALB/c-derived background and a C57Bl/6 background, we are unable to exclude potential differences between mouse strain backgrounds on the assessed inflammatory parameters. As expected, our studies in BALB/c-derived mice had more robust airspace inflammation than studies in C57Bl/6 mice. We are also not able to rule out that differences in the microbiome between animal strains and sources could influence barrier permeability during injury (50). Together, these data demonstrate the complexity of TLR4 function in vivo that must be understood before targeting these pathways to reduce inflammation.

In summary, we used a reductionist model of intratracheal CFH and in vitro testing with cultured and primary alveolar macrophages to determine the role of TLR4 in CFH-induced inflammation. We identified that TLR4 on myeloid cells is important for CFH-dependent cytokine production in the airspace and that TLR4 signaling via MyD88 is a major proinflammatory pathway activated by CFH on macrophages in vitro and ex vivo. These studies show cell-specific activation of inflammatory signaling pathways in response to CFH in the airspace and provide a foundation for future studies aimed at reducing CFH-dependent inflammation as a potential therapeutic intervention for patients with ARDS with elevated airspace CFH.

## DATA AVAILABILITY

Data will be made available upon reasonable request.

## SUPPLEMENTAL DATA

10.6084/m9.figshare.24234268 Supplemental Fig. S1: https://doi.org/10.6084/m9.figshare.24234268.

## GRANTS

This work was supported by NIH Grants HL136888 (to C.M.S.), HL160551 (to C.M.S.), HL126671 (to J.A.B.), HL150783 (to J.A.B.), HL158906 (to L.B.W.), HL164937 (to L.B.W.), GM141927 (to J.K.B.), the National Center for Advancing Translational Sciences Clinical Translational Science Award Program 5UL1TR002243-03, Veteran Affairs I01BX002288 (to J.A.B.), Department of Defense W81XWH-18-1-0683 (to J.A.B.), and the SyBBURE Searle Undergraduate Research Program at Vanderbilt University (to E.Y.Q.).

## DISCLOSURES

Julie Bastarache is an editor of American Journal of Physiology-Lung Cellular and Molecular Physiology and was not involved and did not have access to information regarding the peer-review process or final disposition of this article. An alternate editor oversaw the peer-review and decision-making process for this article. None of the other authors has any conflicts of interest, financial or otherwise, to disclose.

## AUTHOR CONTRIBUTIONS

K.R.S., J.A.B., and C.M.S. conceived and designed research; S.R.L., S.P., E.Y.Q., N.D.P., T.S., A.M.O., and J.K.B. performed experiments; K.R.S., S.R.L., S.P., E.Y.Q., T.S., A.M.O., J.A.B., and C.M.S. analyzed data; K.R.S., S.R.L., L.B.W., J.A.B., and C.M.S. interpreted results of experiments; C.M.S. prepared figures; K.R.S., L.B.W., J.A.B., and C.M.S. drafted manuscript; K.R.S., L.B.W., J.A.B., and C.M.S. edited and revised manuscript; K.R.S., S.R.L., S.P., E.Y.Q., N.D.P., A.M.O., J.K.B., L.B.W., J.A.B., and C.M.S. approved final version of manuscript.
